# Risks and Benefits of Vesicoamniotic Shunting for Lower Urinary Tract Obstruction (LUTO) After Early, Intermediate and Late Onset of Therapy—A Monocentric Study of 104 Treated Cases

**DOI:** 10.3390/biomedicines14010182

**Published:** 2026-01-14

**Authors:** Nadja Riehle, Thomas Kohl

**Affiliations:** 1 German Center for Fetal Surgery and Minimally-Invasive Therapy, University Medical Center Mannheim, 68161 Mannheim, Germany; nadja.riehle@umm.de; 2 Department of Obstetrics and Gynecology, University Medical Center Mannheim, 68161 Mannheim, Germany

**Keywords:** fetus, fetal surgery, prenatal therapy, minimally invasive intervention, LUTO, vesicoamniotic shunting, renal function

## Abstract

**Background/Objectives**: We aimed to assess the risks and benefits of early, intermediate, and late vesicoamniotic shunting (VAS) for lower urinary tract obstruction (LUTO) treated at a single center. **Methods**: A retrospective analysis of 104 fetuses with LUTO that underwent VAS was carried out. The investigation covered the time between the first VAS and postnatal hospital discharge. The cases were analyzed in three groups: Group I fetuses underwent their first intervention ≤ 16 + 0 weeks; Group II fetuses underwent intervention between 16 + 1 and 24 + 0 weeks; and Group III fetuses underwent intervention > 24 + 0 weeks of gestation. Maternal morbidity, pre- and postnatal complications, fetal and neonatal mortality, and urological and renal outcomes were assessed. **Results**: All mothers tolerated the procedures well. Mean gestational age at delivery was 35.4 weeks of gestation. In total, 78 of 104 children were born alive (75%). Postnatal survival was 72 of 78 (92.3%). Overall survival was 72 of 104 (69.2%). Overall, 61.2% of children survived from Group I; 69% of children survived from Group II; and 100% of Group III children survived from the first VAS to postnatal hospital discharge. A total of 41 of 72 survivors (56.9%) were discharged with normal renal function. For 80%, normal renal function was highest after early VAS ≤ 16 + 0 weeks of gestation (Group I), whereas 31% of Group II and 61% of Group III survived with normal renal function. Postnatal pulmonary hypoplasia occurred in 13.3% of the cases of Group I, 40% of Group II, and 23.1% of Group III. **Conclusions**: The findings of this retrospective study confirm that VAS ≤ 16 + 0 weeks of gestation is the best chance for preserved renal function after birth. However, when LUTO is treated later in gestation, fetuses can also benefit from VAS. Pre-interventional sonography may aid in selecting those with the highest chances for a better renal outcome.

## 1. Introduction

Lower urinary tract obstruction (LUTO) is a rare but severe fetal disorder that carries a high risk of perinatal mortality if left untreated [1,2,3,4]. Owing to intrauterine fetal death.

(IUFD) and termination of pregnancy (ToP), several studies have reported live birth rates of less than 20% in pregnancies with untreated fetal LUTO in the first trimester [1,5]. If gestation continues, obstruction mostly leads to progressive kidney damage. The earlier the obstruction occurs, the more severe the dysplastic changes in the kidneys become [6,7,8].

Furthermore, longstanding severe urinary outflow obstruction also leads to pulmonary hypoplasia from low amniotic fluid levels, which is the main cause of neonatal mortality [3,4].

To improve the disease course, efforts have been made for decades to develop prenatal treatment strategies for LUTO. One of the most commonly utilized approaches has been the placement of vesicoamniotic shunts, which aim to decompress the urinary tract and restore amniotic fluid levels [3,9,10,11].

While studies have demonstrated that VAS can improve survival rates by reducing perinatal mortality, evidence regarding its impact on renal function has remained inconclusive for a long time [3,11]. Despite early detection of LUTO, VAS was frequently delayed until after 17 weeks of gestation [3], leading us to assume that the intervention was often too late to prevent irreversible renal damage.

To address this assumption, a new LUTO management approach was introduced at our center more than a decade ago [12]. It focused on initiating treatment for fetuses with severe LUTO within days of the initial diagnosis, starting as early as 12 weeks of gestation. The results of a retrospective data analysis at our center provided early evidence that earlier VAS may lead to better postnatal kidney and pulmonary function: In short, about 80% of fetal patients who had undergone VAS ≤ 16 + 0 weeks of gestation had normal kidney function as neonates. In contrast, fetuses who were treated with VAS between 16 + 1 and 24 + 0 weeks had normal kidney function in about one third of cases postnatally, and if VAS was performed after 24 + 0 weeks, only 20% were born with normal renal function [12]. In the meantime, other centers have achieved and published similarly encouraging outcomes after VAS in the early second trimester [13,14].

However, previous studies have been limited by small sample sizes and marked heterogeneity in terms of inclusion criteria, indications, and timing of intervention, leaving the optimal treatment conditions, peri-interventional prognostic factors, and the specific risks and benefits of VAS at different gestational ages unresolved [12,13,14,15].

These limitations also raise an important question: is shunt placement most therapeutically effective only until the early second trimester, or do additional factors beyond gestational age at diagnosis and intervention also determine prognosis? This issue has gained further relevance as recent findings demonstrate that normal renal function can be preserved in up to 90% of children for 4–10 years after prenatal VAS for LUTO [16]. Identifying the determinants of such favorable long-term outcomes is, therefore, crucial.

The purpose of this study is to provide and discuss perinatal data of 104 patients who underwent VAS for LUTO following our treatment algorithm. This is the largest cohort for the assessment of VAS for LUTO at present. We aim to evaluate the risks and benefits of VAS after early, intermediate, and late intervention and contribute information to identify prognostic factors for the therapy of this complex disease.

## 2. Materials and Methods

The study was conducted in accordance with the Declaration of Helsinki and was approved by the Ethics Committee of the University Medical Center Mannheim (AZ 107/14 & 2019-869R). Informed consent for the study was obtained from all participants.

### 2.1. Patients

Cases were considered eligible for inclusion in the study if, between July 2013 and November 2023, vesicoamniotic shunting for fetal LUTO was performed, a normal karyotype was confirmed, and no sonographic evidence of organ anomalies unrelated to LUTO was observed prior to the first intervention.

Cases were excluded from data analysis if the unborn child had a genomic mutation, organ anomalies unrelated to LUTO were identified before surgery, prenatal treatment with VAS had already been performed at other centers, or if no complete dataset could be obtained because the patient failed to attend post-interventional follow-up appointments.

**Figure 1:**
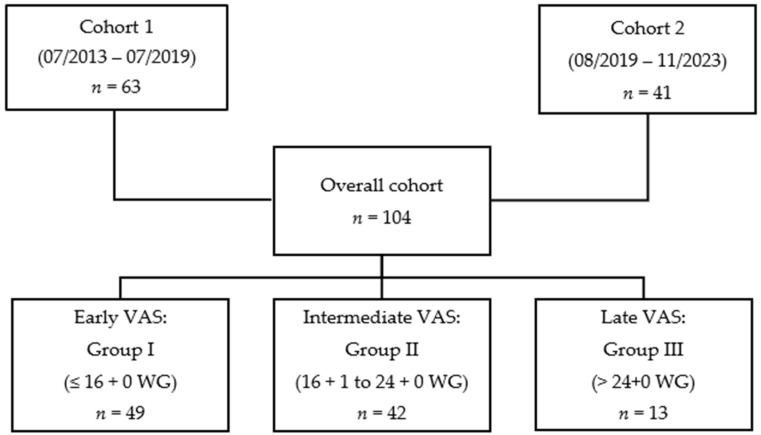
Cohort formation and division into three treatment groups by gestational age at VAS for severe fetal LUTO.

The total cohort for evaluating the perinatal outcomes of fetuses treated with VAS for LUTO comprised 104 cases (Table A1). In total, 63 of these cases were derived from the previously studied and published cohort, which had been treated between July 2013 and July 2019 (Cohort 1) [12]. The additional 41 cases, who were treated between August 2019 and November 2023, formed Cohort 2 (Figure 1).

For retrospective data analysis, both cohorts were pooled and, as in our previous study, divided into three groups (Figure 1): Group I included all children who received the first VAS ≤ 16 + 0 weeks of gestation. Children in Group II were treated between 16 + 1 and 24 + 0 weeks, and Group III comprised those treated from 24 + 1 weeks of gestation onward.

### 2.2. Shunt Insertion

VAS for severe fetal LUTO was performed when the maximum fetal bladder diameter exceeded 25 mm until 14 + 6 weeks of gestation, 30 mm between 15 + 0 and 19 + 6 weeks, and 40 mm from 20 + 0 weeks of gestation onward.

Each surgical procedure was performed employing fetomaternal anesthesia and antibiotic prophylaxis under sterile conditions in an operating room. Through a small skin incision of 3–5 mm on the mother’s abdomen, an ultrasound-guided percutaneous puncture of the dilated fetal bladder was performed using an 18-gauge needle. Preferably, the needle was placed in the lower half of the fetal abdomen below the umbilicus in order to minimize the need for repeated shunt placement. Then, either a pigtail catheter (Cook Deutschland GmbH, Mönchengladbach, Germany) or a Somatex stent shunt (Intrauterine shunt, Somatex Medical Technologies, Berlin, Germany) was placed as a vesicoamniotic shunt. Within the same intervention, amniotic fluid or fetal urine was sampled in order to perform fetal karyotyping.

One day after surgery, another ultrasound examination was performed to verify fetal vitality and the position of the shunt. If no complications, such as preterm, premature rupture of membranes (PPROMs), chorioamnionitis, or shunt displacement, were detected, the patient was discharged from inpatient care. Subsequently, weekly outpatient follow-up examinations were performed until the end of the gestation to detect any recurrence of urinary obstruction early.

In cases of recurrent urinary tract obstruction, which typically occurred due to shunt occlusion, shunt displacement as a result of fetal growth, or additional obstruction of the upper urinary tract, immediate reintervention in the form of a vesicoamniotic, ureteroamniotic, or renoamniotic shunt was scheduled to prevent further kidney damage.

In cases where anhydramnios was present 30 weeks before gestation, serial amniotic fluid infusions and, if necessary, fetoscopic endoluminal tracheal occlusion (FETO) were offered to the mother to prevent or treat severe pulmonary hypoplasia.

### 2.3. Data Analysis

Perinatal outcome data for Cohort 1, comprising cases treated between July 2013 and July 2019, had already been reported in our previous study [12]. Data for all subsequent cases treated between August 2019 and November 2023 were extracted retrospectively from the digital patient management system of the University Medical Center Mannheim.

The analysis included sonographic images and results from prenatal diagnostics, genetic analyses, surgical reports from prenatal interventions, birth records, postnatal laboratory results, and reports on the postnatal treatment course up to the point of discharge.

The following parameters were assessed: gestational age at the time of the first intervention; type and number of re-interventions; gestational age at the time of the first re-intervention; maternal morbidity and mortality; occurrence of IUFD, ToP, PPROM, chorioamnionitis and live birth rate; gestational age at delivery; mode of delivery; postnatal pulmonary hypoplasia; postnatal mortality; and postnatal renal function.

To assess renal function, creatinine, urea, and electrolyte levels from blood tests were assessed. The blood work was taken at variable time points after birth, depending on the length of hospitalization. Furthermore, the need for nephrological treatment was recorded, particularly whether dialysis was performed.

“Normal renal function” was assigned to children with normal urine production and age-related laboratory values for blood urea, creatinine, and electrolytes without requiring chronic nephrological therapy.

“Impaired renal function” was diagnosed when serum creatinine remained elevated, electrolytes were imbalanced, or nephrological treatment was required, though without the need for renal replacement therapy.

“End-stage renal disease” required renal replacement therapy, such as hemodialysis or peritoneal dialysis.

In neonates discharged within the first few days of life, transiently elevated creatinine values of up to 0.77 mg/dL were observed, most likely reflecting residual maternal creatinine rather than impaired neonatal renal function. As these values normalized during subsequent follow-up examinations and renal function remained normal throughout long-term follow-up of up to ten years in 90% of these cases, renal function was classified as normal despite the initial transient elevation [16].

### 2.4. Statistics

Amniotic infection rates, postnatal mortality, pulmonary hypoplasia, and renal function were compared between the three treatment groups using Fisher’s exact test. The Chi^2^ test was applied to assess differences in prenatal mortality, the occurrence of PPROM, the frequency of TOP and IUFD, preterm delivery, the need for re-interventions, and overall survival across Groups I–III. Associations between therapy group and either gestational age at birth or number of interventions per fetus were analyzed using the Kruskal–Wallis test. To evaluate the impact of the number of prenatal interventions on gestational age at birth, the Mann–Whitney U test was performed.

## 3. Results

### 3.1. Vesicoamniotic Shunting

In total, 49 cases were assigned to Group I (first VAS ≤ 16 + 0 weeks of gestation). The first intervention was performed between 12 + 0 and 16 + 0 weeks in this group. The mean gestational age at the time of the intervention was 14.3 weeks (Table 1).

In total, 42 cases were classified as Group II (first VAS between 16 + 1 and 24 + 0 weeks of gestation). The initial intervention took place between 16 + 1 and 23 + 6 weeks. The mean gestational age at the time of first intervention was 18.4 weeks (Table 1).

In total, 13 cases were included in Group III (first VAS > 24 + 0 weeks of gestation). In this group, the first shunt insertion was performed between 25 + 6 and 32 + 0 weeks. The mean gestational age at the time of first intervention was 29.3 weeks (Table 1).

In total, 158 shunt insertions were carried out, ranging from one to four procedures per fetus. In 66 fetuses (63.5%), treatment consisted of a single intervention, whereas 38 fetuses (36.5%) required more than one procedure, resulting in a mean intervention frequency of 1.52 procedures per fetus. The frequency of re-interventions varied across groups: 32.7% of fetuses in Group I required ≥2 interventions, compared with 42.9% in Group II and 30.8% in Group III (Table 1). The average number of interventions was 1.5, 1.6, and 1.4 in Groups I, II, and III, respectively. No significant association was found between therapy group and frequency of re-interventions, indicating that earlier shunt placement did not increase the need for reintervention (*p* = 0.73).

### 3.2. Other Prenatal Surgical Measures

A total of 88 fetuses (84.6%) underwent vesicoamniotic shunt placement only, while in 16 cases (15.4%) additional peritoneoamniotic, renoamniotic, or ureteroamniotic shunts were inserted—either during the initial VAS procedure or as a reintervention due to recurrent or persistent urinary obstruction.

Five fetuses in Group I (10.2%), three in Group II (7.1%), and three in Group III (23.1%) received shunting of the upper urinary tract (Table 2). In two cases in Group I (4.1%), three cases in Group II (7.1%), and four cases in Group III (30.8%), peritoneoamniotic shunting was performed due to urinary ascites after shunt dislodgement after VAS (Table 2).

In cases with anhydramnios before 30 weeks of gestation, serial amnioinfusions were performed in seven cases of Group II (16.7%), three cases of Group I (6.1%), and one case of Group III (7.7%) to prevent pulmonary hypoplasia.

Two fetuses in Group II (4.8%) showed prenatal signs of severe pulmonary hypoplasia and, therefore, underwent FETO. No fetus in Groups I or III was treated with FETO (Table 2).

### 3.3. Prenatal Complications and Delivery

Although all fetuses were alive at the end of surgery, 11 out of 104 fetuses (10.4%) died spontaneously in utero during the course of pregnancy. Six IUFDs occurred within hours to days after VAS. The remaining five fetuses died one to eleven weeks post-procedure. The exact causes of IUFD, however, could not be determined.

The frequency of IUFDs differed significantly between the therapy groups (*p* = 0.01). Ten intrauterine fetal deaths occurred in Group I (20.4%), one death occurred in Group II (2.3%), and no deaths occurred in Group III (0%) (Table 1). Fifteen women opted for termination of pregnancy—nine in Group I (18.4%) and six in Group II (14.3%). No termination of pregnancy was performed in Group III (0%).

The peri-interventional infection rate was 0%. However, during the course of pregnancy, complications such as PPROM or amniotic infection occurred in 17 cases (16.3%) (Table 1). In two cases, PPROM was accompanied by amniotic infection, while in five additional pregnancies, the infection developed without PPROM. PPROM occurred in three pregnancies in Group I (6.1%), seven pregnancies in Group II (16.7%), and three pregnancies in Group III (23.1%). While PPROM was more common in Group II and Group III compared to Group I, the risk of amniotic infection was highest in Group I (8.2% vs. 4.8% vs. 6.7%). However, no significant association was found between the occurrence of amniotic infection and the three therapy groups (*p* = 0.76) or between the occurrence of PPROM and the therapy groups (*p* = 0.15).

**Figure 2:**
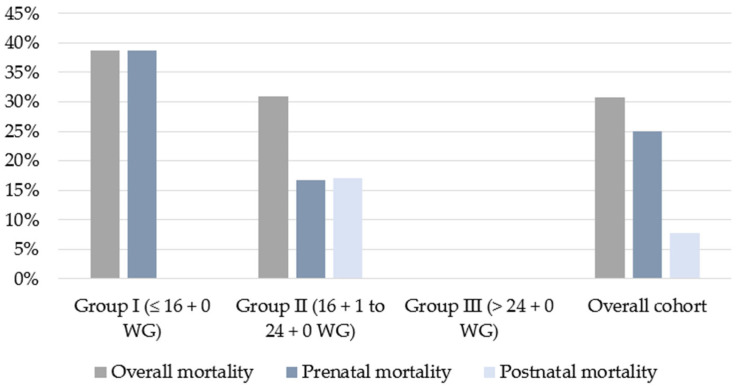
Pre-, postnatal, and overall mortality in fetuses with LUTO treated with VAS, comparing early, intermediate, and late intervention (Groups I–III).

After prenatal mortality caused by IUFD and ToP, 78 out of 104 fetuses were born alive (75%). A total of 30 fetuses in Group I survived until birth (61.2%), 35 fetuses survived until birth in Group II (83.3%), and 13 fetuses survived until birth in Group III (100%) (Figure 2).

The earliest delivery occurred at 27 + 1 weeks of gestation due to amniotic infection, while the latest delivery occurred at 40 + 0 weeks. In total, 68 children were delivered by cesarean section (87.2%), and 10 were delivered spontaneously (12.8%). The overall mean gestational age at delivery was 35.4 weeks of gestation. Neither the gestational age at the time of birth nor the risk for premature delivery significantly differed between the early, intermediate, or late therapy groups. The mean gestational ages at delivery were 35.6 weeks for Group I, 35.4 weeks for Group II, and 35.2 weeks for Group III (*p* = 0.66) (Table 3).

In Group I, 19 children were born prematurely (63.3%), followed by 27 children in Group II (77.1%) and nine children in Group III (69.2%) (*p* = 0.47). Of the 78 live-born children (75%), 21 were born with pulmonary hypoplasia (26.9%). Of these, 4 children belonged to Group I, 14 children belonged to Group II, and three belonged to Group III. Hence, children who received early shunt therapy ≤16 + 0 weeks of gestation had a lower risk for pulmonary hypoplasia (13.3%) compared to those in Group II (40%) or Group III (23.1%), (*p* = 0.05). Furthermore, especially in Group II, pulmonary hypoplasia was frequently more severe than in the other groups, with six children dying in connection with the condition. No children with pulmonary hypoplasia died in either Group I or Group III.

### 3.4. Mortality

Overall, prenatal mortality due to termination of pregnancy and IUFDs was 25%. Since the highest rate of terminations of pregnancies occurred in Group I and more fetuses died after VAS compared to other therapy groups, Group I showed the highest prenatal mortality rate at 38.8%. In Group II, the prenatal mortality rate was 23.3%, and in Group III, it was 0%. The correlation between earlier therapy and increased prenatal mortality was statistically significant (*p* = 0.004), (Figure 2).

Postnatally, neonatal death occurred in only 6 of 78 cases (7.7%). All children in Group I and Group III survived the neonatal period until hospital discharge. By contrast, the postnatal mortality in Group II was significantly higher, with six children (17.1%) from this group dying after birth (*p* = 0.02). Neonatal death was associated with pulmonary hypoplasia in all six cases (Figure 2).

As a result of the postnatal mortality rate of 17.1% in Group II, the overall mortality rate in Group I approximated that of Group II during the first few weeks of life. The total mortality rate was 38.8% in Group I and 31.0% in Group II. By contrast, no deaths occurred in Group III, either prenatally or postnatally, resulting in a total mortality rate of 0% among fetuses treated with VAS after 24 + 0 weeks of gestation (Table 3).

Overall, 72 children survived (69.2%) from the first prenatal intervention to discharge after birth. Survival was achieved in 30 cases (61.2%) in Group I; 29 cases (69.0%) in Group II; and all 13 cases (100%) in Group III. Among the survivors, 6 were female (8.3%), and 66 were male (91.7%).

### 3.5. Postnatal Renal Function

**Figure 3:**
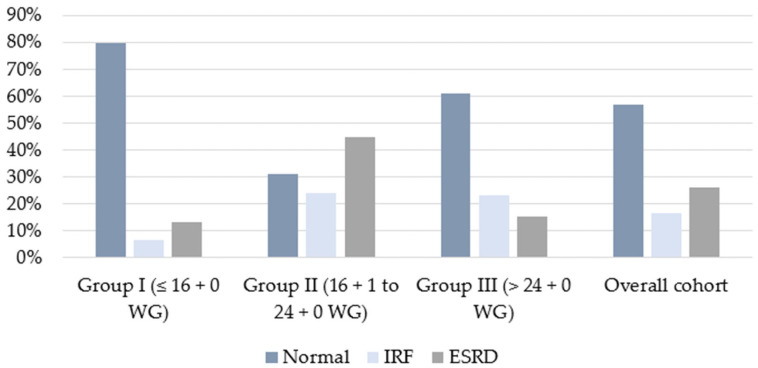
Postnatal renal function in children with LUTO treated with VAS, comparing early, intermediate, and late intervention (Groups I–III).

Renal function was evaluated for all children who survived until hospital discharge (*n* = 72). In total, 41 of 72 survivors (56.9%) were discharged with normal renal function, 12 (16.7%) were discharged with impaired renal function, and 19 (26.3%) were discharged with end-stage renal disease (Figure 3). There was a significant difference in postnatal renal function between the early, intermediate, and late treatment groups (*p* = 0.002). While 24 children (80%) with early prenatal therapy ≤16 + 0 weeks of gestation (Group I) were born with normal renal function, only 9 children in Group II (31%), who received prenatal therapy between 16 + 1 and 24 + 0 weeks, were born with normal renal function (Figure 3, Table 3). 

In Group III, where fetal surgery for LUTO was performed after 24 + 0 weeks, eight children (61%) showed normal renal function postnatally. Impaired renal function was present in two children in Group I (6.7%), seven children in Group II (24.1%), and three children in Group III (23.1%). End-stage renal disease requiring postnatal dialysis developed in 13 cases of Group II (44.8%) and in 2 cases of Group III (15.4%). Only four children from Group I (13.3%) required such treatment (Table 3, Figure 3).

### 3.6. Underlying Pathology of LUTO

At our center, postnatal cystoscopy is usually performed several months after birth, as the urethra in children with LUTO is often too small to allow cystoscopy during the first few weeks of life [17]. As differentiation between various intraurethral anomalies was not feasible before hospital discharge, in the absence of cystoscopy, the differential diagnoses of PUV, urethral stenosis, and urethral hypoplasia were grouped under the term isolated urethral malformation for the purpose of this analysis.

Among the 72 survivors, isolated urethral malformation (IUM) was the most common cause for LUTO, accounting for 55 children (76.4%). Urethral atresia was responsible for 8.3% of the obstructions, with one case presenting as isolated urethral atresia and the other five being associated with complex cloacal malformations. Additionally, there was one case each of ureterocele, labial synechia, abdominal tumor, and prune-belly syndrome. In seven cases (9.7%), the cause of LUTO could not be determined postnatally, leaving it unknown.

**Figure 4:**
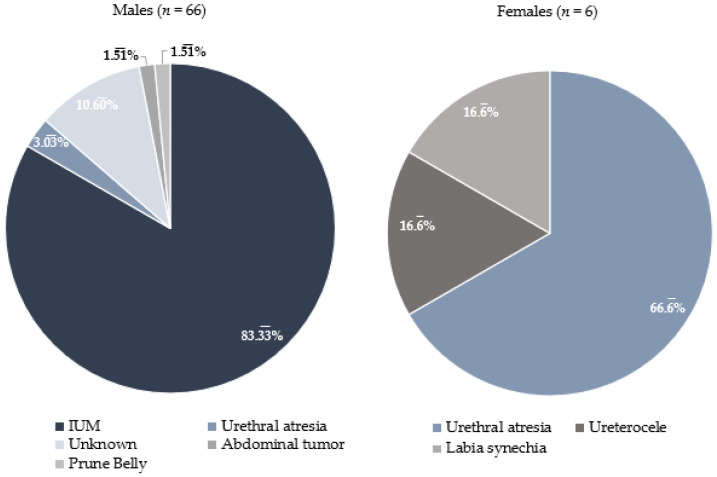
Etiology of LUTO in male and female neonatal survivors after VAS.

However, the underlying cause of LUTO differed significantly between males and females (Figure 4). In 55 male survivors (83.3%), LUTO was caused by IUM. Only one case involved a complex ano-uro-genital malformation with urethral atresia. Additionally, one case each was caused by an abdominal tumor (neuroblastoma) and prune-belly syndrome. In surviving female infants, cloacal malformation with urethral atresia was the most frequent cause of LUTO (66.7%). However, in two female children (33.3%), the obstruction was not caused by complex malformations but by a total labial synechia or ureterocele.

The analysis also investigated whether the cause of LUTO correlates with postnatal renal outcomes. The results indicate no statistically significant association between normal renal function and either isolated urethral malformation or complex malformation (*p* = 0.42).

## 4. Discussion

The optimal timing for VAS still remains controversial [18]. Critics of early intervention argue that VAS in the first trimester is associated with a higher risk of complications such as chorioamnionitis, PPROM, and preterm delivery. Nevertheless, our study found that associated risks of VAS, such as the occurrence of chorioamnionitis or PPROM and the impact on preterm delivery, did not differ significantly between the early, intermediate, and late intervention Groups I–III. Therefore, no clear advantage or disadvantage can be derived for one specific treatment group with regard to these complications.

However, differences between the therapy groups could be found for pre- and postnatal mortality, overall survival, and postnatal kidney function. In contrast to Groups II and III, in which intervention was performed after 16 + 0 weeks of gestation, Group I showed higher post-interventional prenatal mortality, primarily attributable to ToP (18.4%) and IUFDs (20.4%). The high number of IUFDs in Group I may in part be attributable to the intervention itself. Although no procedure was complicated by immediate bleeding or PPROM, and all fetuses were viable at the end of surgery, half of the IUFDs occurred within the first day following VAS, while the other half occurred weeks or months after surgery. In the former cases, we hypothesize that kinking of the umbilical vessels may have contributed to early fetal demise. Due to their course and connection to the enlarged bladder, the umbilical vessels become markedly elongated; after bladder decompression, the rapid reduction in bladder size abruptly decreases this elongation, which may lead to luminal occlusion from vessel kinking. This effect is likely more pronounced in early gestation when bladder diameter is disproportionately larger relative to overall fetal size compared with later stages of pregnancy. Along this line of reasoning, we speculate that fetal demise before intervention may be caused by the same pathophysiological mechanism—impaired fetoplacental blood flow resulting from progressive narrowing of the umbilical vessels with growing bladder size.

Nonetheless, the remaining half of IUFDs occurred up to eleven weeks after the intervention, making a causal link to the early procedure unlikely. In these cases, undetected organ anomalies or genetic disorders may have contributed. Since detailed organ ultrasound screening was not feasible at ≤16 + 0 weeks of gestation, associated anomalies were more frequently undiagnosed prior to the procedure. By contrast, in Groups II and III, sonographic organ screening was more feasible, enabling the identification of additional malformations before intervention. Consequently, more fetuses with undetected anomalies or genetic disorders might have been treated in Group I, leading to a higher post-interventional rate of IUFDs than in Groups II and III. A similar rationale may explain the higher number of ToPs in Group I. In our clinical experience, mothers choose ToP when complex malformation or signs of renal failure are present. In Group I, these findings were mostly detected at a later stage, leading to ToP after VAS. In fetuses where LUTO was diagnosed at >16 weeks of gestation, such decisions were more commonly made prior to the intervention. Because ToPs or IUFDs that occurred prior to the intervention are not represented in our study, this might explain why more IUFDs and terminations of pregnancy were observed in Group I.

Although early intervention was associated with an increased risk of prenatal mortality, the potential benefits appear to outweigh these risks, given the favorable postnatal outcomes. Significantly fewer neonates in Group I (VAS ≤ 16 + 0 weeks of gestation) were born with pulmonary hypoplasia and died from respiratory failure, as early bladder decompression allowed better lung development than in the intermediate or late therapy groups. Consequently, overall mortality in Group I gradually aligned with that of Group II during the postnatal period. Furthermore, even the high prenatal mortality in Group I remained substantially lower than the prenatal mortality reported in the literature for untreated fetuses with LUTO, which has been described to reach rates of up to 80% [1,5].

Early VAS also provided the highest likelihood of preserved renal function, with 80% of children in Group I born with normal renal function. Since fetal urine production does not begin before 12 weeks of gestation, obstruction before 16 + 0 weeks seems unlikely to persist long enough to cause end-stage renal disease. In contrast, delaying therapy beyond 17 weeks, as in Group II, was associated with a markedly lower rate of preserved renal function (31%) and higher neonatal mortality (17%) due to pulmonary hypoplasia.

At first glance, the renal outcomes in Group III appear to contradict the findings in Groups I and II. Based on our previous study [12], one would expect an even lower likelihood of preserved renal function due to the later timing of intervention. However, the observed 63% rate of preserved renal function in Group III reflects better pre-interventional selection, introducing a degree of sampling bias. In late pregnancy, sonographic findings and clinical presentations of LUTO are highly variable, which makes it more challenging to define which fetuses may benefit the most from VAS. On the other hand, detailed fetal organ sonography is feasible in the late second and third trimester, allowing prognostic factors for renal dysplasia to be assessed more accurately.

Notably, we observed that most mothers in Group III with normal postnatal renal function reported that routine ultrasound examinations within a month before diagnosis had shown no signs of megacystis. In these cases, it can be inferred that the obstruction developed more recently. This observation of a later LUTO was supported by the presence of sufficient lung development despite an impaired amount of amniotic fluid. These favorable prognostic features influenced parental decisions to proceed with VAS. Conversely, when sonography indicated longstanding LUTO—characterized by oligohydramnios, cortical cysts, increased renal echogenicity, and pulmonary hypoplasia—the chance of a successful outcome was considered low. In such cases, parents often declined prenatal therapy, excluding these fetuses from the study.

Overall, our analysis indicates that the risks and benefits of VAS depend not only on gestational age at the time of intervention but also on additional factors, which can be defined by detailed sonography. This leads to the following conclusions:

Before 16 weeks of gestation, fetuses generally have the best chance of benefitting from immediate VAS, even without accurate pre-interventional selection, as urinary tract obstruction at this stage typically has not persisted long enough to cause significant renal damage. However, since detailed sonographic organ screening is not yet feasible at this gestational age, it should be emphasized during counseling that further malformations may become apparent after the intervention, and the combined risks of IUFD and ToP are approximately 38%. Nevertheless, 62% of children who survive the pregnancy generally show preserved renal function in 80% of cases and usually survive the neonatal period. Moreover, a follow-up examination of these children has shown that preserved renal function at the time of delivery remains normal for at least 4–10 years [16]. However, when LUTO remains untreated for 6–8 weeks, the affected children do not benefit from VAS in the late second or third trimester due to irreversible renal damage, as they show high risk for end-stage renal disease, pulmonary hypoplasia, and postnatal demise.

Conversely, severe LUTO can also develop in the late second or third trimester. In such cases, if the period of obstruction is less than approximately four weeks, timely intervention may still provide a substantial benefit. Therefore, the better renal outcomes observed in Group III compared to Group II do not imply that intervention should be routinely delayed until 24 weeks in cases of early-onset LUTO. Rather, the results highlight that urogenital tract malformations can cause significant obstruction later in pregnancy, and that affected fetuses may still benefit from immediate intervention in this situation; thus, fetal selection is necessary to identify these cases.

However, the precise analysis of sonographic parameters and their influence on fetal and neonatal prognosis is limited by the retrospective design of this study and cannot be fully addressed here. To close this gap, prospective research is warranted to determine which sonographic findings serve as reliable prognostic indicators and how they should guide therapeutic decisions.

### Limitations

Our study is limited by its retrospective design. Furthermore, our study has a relatively short follow-up period, covering only the time between the first prenatal intervention and postnatal hospital discharge of the affected neonates. Although there is evidence that neonatally preserved renal function after VAS for LUTO can be maintained long-term [16], the sustainability of our therapeutic approach requires further investigation.

## Figures and Tables

**Table 1:** Prenatal characteristics of the early, intermediate, and late treatment groups (Groups I–III).

	Group I (≤16 + 0 WG) *n* = 49	Group II (16 + 1 to 24 + 0 WG) *n* = 42	Group III (>24 + 0 WG) *n* = 13	Significance (*p* = 005)
∅ GA at 1. intervention	14.3	18.4	29.3	
∅ Interventions per fetus	1.45	1.64	1.38	*p* = 0.73
PPROM	3 (6.1%)	7 (16.7%)	3 (23.1%)	*p* = 0.15
Chorioamnionitis	4 (8.2%)	2 (4.8%)	1 (6.7%)	*p* = 0.76
ToP	9 (18.4%)	6 (14.3%)	0	
IUFD	10 (20.4%)	1 (2.3%)	0	*p* = 0.01
Overall prenatal mortality	19 (38.8%)	7 (16.7%)	0	*p* = 0.004

∅ = mean (average).

**Table 2:** Prenatal surgical measures apart from VAS in the early, intermediate, and late treatment groups (Groups I–III).

	Group I (≤16 + 0 WG) *n* = 49	Group II (16 + 1 to 24 + 0 WG) *n* = 42	Group III (>24 + 0 WG) *n* = 13
Uretero- or renoamniotic shunt	5 (10.2%)	3 (7.1%)	3 (23.1%)
Peritoneoamniotic shunt	2 (4.1%)	3 (7.1%)	4 (30.8%)
Amnioinfusion	3 (6.1%)	7 (16.7%)	1 (7.7%)
FETO	0	2 (4.8%)	0

**Table 3:** Postnatal characteristics of live-born children of the early, intermediate, and late treatment groups (Groups I–III).

	Group I (≤16 + 0 WG)	Group II (16 + 1 to 24 + 0 WG)	Group III (>24 + 0 WG)	Significance (*p* = 0.05)
	*n* = 30	*n* = 35	*n* = 13	
∅ GA at delivery	35.6	35.4	35.2	*p* = 0.66
Preterm deliveries	17 (56.7%)	27 (77.1%)	9 (69.2%)	*p* = 0.47
Pulmonary Hypoplasia	4 (13.3%)	14 (40%)	3 (23.1%)	*p* = 0.05
Postnatal mortality	0 (0%)	6 (17.1%)	0 (0%)	*p* = 0.02
	*n* = 30	*n* = 29	*n* = 13	
Normal renal function	24 (80%)	9 (31%)	8 (61%)	*p* = 0.002
IRF	2 (6.7%)	7 (24.1%)	3 (23.1%)	
ESRD	4 (13.3%)	13 (44.8%)	2 (15.4%)	

∅ = mean (average).

## Data Availability

The original contributions presented in this study are included in the article. Further inquiries can be directed to the corresponding author.

## Abbreviations

The following abbreviations are used in this manuscript:

ESRD	End-stage renal disease
FETO	Fetoscopic endoluminal tracheal occlusion
GA	Gestational age
IUFD	Intrauterine fetal death
IUM	Isolated urethral malformation
IRF	Impaired renal function
LUTO	Lower urinary tract obstruction
PPROM	Preterm, premature rupture of membranes
ToP	Termination of pregnancy
VAS	Vesicoamniotic shunting
WG	Weeks of gestation

## Appendix A

**Table A1:** Perinatal characteristics of 104 cases after early, intermediate, and late VAS (Groups I–III) for severe fetal LUTO.

Case No. #	f/m	GA 1. VAS	No. of Interventions	Prenat. Complications	GA Birth	Mode of Delivery	PH	Outcome Survival	Postnat. Renal Function	Crea (mg/dL)	Cause of LUTO
**Group I (VAS ≤ 16 + 0 WG)**											
1	f	13 + 1	3	-	32 + 6	CS	-	survivor	normal	0.54	cloaca, UA
2	m	13 + 1	3	-	39 + 4	Spont.	-	survivor	normal	0.77	IUM
3	m	13 + 2	2	-	32 + 0	CS	-	survivor	normal	0.4	cloaca, UA
4	m	14 + 1	2	chorioamnionitis	35 + 4	CS	-	survivor	normal	0.2	IUM
5	m	14 + 1	2	-	35 + 4	CS	-	survivor	normal	0.3	IUM
6	m	14 + 1	3	chorioamnionitis	27 + 1	CS	+	survivor	normal	0.3	Prune-Belly
7	m	15 + 2	2	-	33 + 1	CS	-	survivor	normal	0.2	IUM
8	f	15 + 2	2	-	35 + 2	CS	-	survivor	normal	0.4	cloaca, UA
9	m	15 + 5	1	-	30 + 1	CS	-	survivor	normal	0.3	IUM
16	m	14 + 2	3	-	37 + 1	Spont.	-	survivor	IRF	0.8	IUM
17	m	15 + 4	1	-	37 + 2	Spont.	-	survivor	IRF	2.1	u
25	m	14 + 3	1	chorioamnionitis	34 + 4	CS	+	survivor	ESRD	2.19	IUM
26	m	14 + 3	3	chorioamnionitis	34 + 4	CS	-	survivor	ESRD	2.17	IUM
38	m	13 + 2	1	-				IUFD			
39	m	13 + 4	1	-				IUFD			
40	m	14 + 0	2	-				ToP			
41	m	14 + 3	1	-				IUFD			
42	m	14 + 4	1	-				ToP			
43	m	15 + 2	1	-				ToP			
50	m	15 + 6	2	-	38 + 1	CS	-	survivor	normal	0.18	IUM
53	m	14 + 2	1	-				ToP			
54	m	15 + 5	1	PPROM	36 + 2	CS	-	survivor	normal	0.22	IUM
55	m	14 + 4	1	-				ToP			
58	m	15 + 0	1	-	38 + 0	CS	-	survivor	normal	0.3	IUM
59	f	14 + 4	1	-	37 + 0	CS	-	survivor	normal	0.49	cloaca, UA
60	m	12 + 5	1	-	37 + 6	CS	-	survivor	normal	0.31	IUM
61	m	14 + 6	1	-				ToP			
63	m	13 + 5	1	-	33 + 1	CS	-	survivor	normal	0.35	IUM
71	m	13 + 1	1	-				IUFD			
72	m	13 + 6	1	PPROM	35 + 5	CS	+	survivor	ESRD	3.19	IUM
73	m	15 + 4	2	-	36 + 0	CS	-	survivor	normal	0.22	IUM
74	m	15 + 5	2	PPROM	31 + 6	CS	+	survivor	ESRD	4.99	u
75	m	14 + 1	1	-				ToP			
77	u	14 + 2	1	-				IUFD			
79	m	14 + 2	1	-	36 + 2	CS	-	survivor	normal	0.23	IUM
80	u	15 + 2	1	-				IUFD			
81	m	14 + 0	3	-	38 + 4	CS	-	survivor	normal	0.21	IUM
82	m	13 + 2	1	-	35 + 2	CS	-	survivor	normal	0.21	IUM
83	f	12 + 6	1	-				IUFD			
86	f	14 + 1	1	-				ToP			
87	m	14 + 3	1	-				ToP			
88	m	15 + 5	1	-	38 + 4	Spont.	-	survivor	normal	0.22	IUM
89	m	13 + 6	1	-	39 + 1	Spont.	-	survivor	normal	0.37	IUM
91	f	14 + 0	1	-				ToP			
92	m	12 + 0	1	-				IUFD			
96	m	14 + 0	1	-				IUFD			
98	f	12 + 6	1	-	37 + 5	CS	-	survivor	normal	0.3	cloaca, UA
100	m	14 + 3	2	-	34 + 3	CS	-	survivor	normal	0.59	IUM
101	m	13 + 4	1	-	38 + 3	CS	-	survivor	normal	0.18	IUM
**Group II (VAS 16 + 1 to 24 + 0 WG)**											
10	m	16 + 4	1	-	35 + 4	CS	-	survivor	normal	0.2	IUM
11	m	17 + 2	3	-	31 + 0	CS	-	survivor	normal	0.4	IUM
12	m	19 + 4	1	-	35 + 1	CS	-	survivor	normal	0.4	IUM
13	m	18 + 5	3	PPROM	32 + 0	CS	-	survivor	normal	0.45	IUM
14	m	18 + 5	2	-	35 + 2	CS	+	survivor	normal	0.3	IUM
18	m	16 + 4	1	-	38 + 0	CS	-	survivor	IRF	0.6	IUM
19	m	18 + 5	3	-	35 + 2	CS	-	survivor	IRF	0.7	IUM
20	m	18 + 4	2	-	34 + 2	CS	-	survivor	IRF	0.9	UA
24	m	20 + 5	3	-	35 + 1	CS	-	survivor	IRF	1.2	IUM
27	m	16 + 1	3	PPROM, chorio-amnionitis	32 + 5	Spont.	-	survivor	ESRD	3.2	u
28	m	18 + 1	2	-	37 + 6	CS	-	survivor	ESRD	2.45	u
29	m	16 + 4	3	PPROM, chorio-amnionitis	33 + 4	CS	-	survivor	ESRD	2.13	IUM
30	m	17 + 3	1	-	36 + 5	CS	-	survivor	ESRD	1.15	IUM
31	m	18	1	-	38 + 0	Spont.	+	survivor	ESRD	3.1	u
32	m	18	1	-	38 + 0	CS	+	survivor	ESRD	2.3	IUM
33	m	18 + 3	1	-	36 + 6	Spont.	-	survivor	ESRD	6	u
34	m	19	1	-	34 + 2	CS	+	survivor	ESRD	3.2	IUM
35	m	20	3	-	38 + 2	CS	+	survivor	ESRD	2.45	u
37	m	23 + 6	2	-	35 + 2	CS	+	neonatal loss			
44	m	16 + 1	1	PPROM				ToP			
45	m	16 + 2	2	PPROM	30 + 0	CS	+	neonatal loss			
46	m	16 + 3	3	-				ToP			
47	m	18 + 5	1	-	27 + 6	CS	+	neonatal loss			
48	m	20 + 4	1	-				ToP			
49	m	22 + 4	1	-				ToP			
51	m	17	1	-	35 + 5	CS	-	survivor	normal	0.36	IUM
52	f	18 + 1	2	-	37 + 5	CS	-	survivor	normal	0.5	Ureterocele
56	m	21 + 4	2	-	33 + 2	CS	-	survivor	normal	0.45	IUM
57	m	21	1	-	37 + 2	CS	-	survivor	IRF	1.65	IUM
62	m	17	2	-	34 + 2	CS	-	survivor	IRF	1.4	IUM
64	m	18 + 4	3	PPROM	36 + 2	CS	-	survivor	IRF	1.03	IUM
67	m	16 + 3	1	-	34 + 4	CS	-	neonatal loss			
68	m	16 + 6	1	-	35 + 5	CS	+	neonatal loss			
70	m	17 + 4	1	-	39 + 1	CS	+	survivor	ESRD	u	IUM
76	m	20 + 3	1	-	35 + 5	CS	+	survivor	ESRD	3.29	IUM
78	m	17 + 2	1	-				ToP			
85	f	20 + 2	1	-				ToP			
90	m	16 + 1	1	-	35 + 1	CS	+	survivor	ESRD	2.5	IUM
95	m	18 + 0	1	-	36 + 5	CS	-	survivor	normal	1.55	IUM
97	f	16 + 3	1	-				IUFD			
102	m	17 + 3	1	-	36 + 5	CS	-	survivor	ESRD	2.88	IUM
103	m	18 + 0	2	PPROM	34 + 4	CS	+	neonatal loss			
**Group III (VAS > 24 + 0 WG)**											
15	m	30 + 3	2	-	34 + 2	CS	-	survivor	normal	0.54	IUM
21	m	28 + 5	1	-	39 + 1	Spont.	-	survivor	IRF	2.09	IUM
22	m	28 + 4	1	-	40 + 0	CS	-	survivor	IRF	1.2	IUM
23	m	26 + 5	3	-	34 + 6	CS	-	survivor	IRF	0.6	IUM
36	m	26 + 1	1	-	35 + 5	CS	+	survivor	ESRD	2.1	IUM
65	m	27 + 6	1	PPROM	29 + 2	CS	-	survivor	normal	0.18	IUM
66	m	32 + 0	2	-	37 + 5	Spont.	-	survivor	normal	0.15	IUM
69	m	29 + 3	1	PPROM	32 + 6	CS	-	survivor	normal	0.55	IUM
84	f	25 + 6	1	-	38 + 0	Spont.	-	survivor	normal	0.8	Labia-synechia
93	m	26 + 3	1	PPROM	33 + 4	CS	+	survivor	ESRD	2.51	IUM
94	m	32 + 0	2	chorioamnionitis	33 + 1	CS	-	survivor	normal	0.83	IUM
99	m	34 + 4	1	-	35 + 3	CS	+	survivor	normal	0.31	IUM
104	m	31 + 4	1	-	33 + 2	CS	-	survivor	normal	0.15	Abdominal tumor

Abbreviations: Crea = creatinine; CS = cesarean section; ERSD = end-stage renal disease; f = female; GA = gestational age; IRF = impaired renal function; IUFD = intrauterine fetal death; IUM = isolate urethral malformation; No. = number; m = male; PH = pulmonary hypoplasia; PPROMs = preterm premature rupture of membranes; spont.= spontaneous delivery; ToP = termination of pregnancy; u = unknown; UA = urethral atresia; VAS = vesicoamniotic shunting; WG = weeks of gestation; + = yes; - = no; # = case number.
